# Cattle with a precise, zygote-mediated deletion safely eliminate the major milk allergen beta-lactoglobulin

**DOI:** 10.1038/s41598-018-25654-8

**Published:** 2018-05-16

**Authors:** Jingwei Wei, Stefan Wagner, Paul Maclean, Brigid Brophy, Sally Cole, Grant Smolenski, Dan F. Carlson, Scott C. Fahrenkrug, David N. Wells, Götz Laible

**Affiliations:** 1 AgResearch, Ruakura Research Centre, Hamilton, 3240 New Zealand; 2 Rowett Institute, Aberdeen, AB25 2ZD United Kingdom; 3 MS3 Solutions Ltd., Ruakura Research Centre, Hamilton, 3240 New Zealand; 4 Recombinetics, St. Paul, MN United States

## Abstract

We applied precise  zygote-mediated genome editing to eliminate beta-lactoglobulin (BLG), a major allergen in cows’ milk. To efficiently generate LGB knockout cows, biopsied embryos were screened to transfer only appropriately modified embryos. Transfer of 13 pre-selected embryos into surrogate cows resulted in the birth of three calves, one dying shortly after birth. Deep sequencing results confirmed conversion of the genotype from wild type to the edited nine bp deletion by more than 97% in the two male calves. The third calf, a healthy female, had in addition to the expected nine bp deletion (81%), alleles with an in frame 21 bp deletion (<17%) at the target site. While her milk was free of any mature BLG, we detected low levels of a BLG variant derived from the minor deletion allele. This confirmed that the nine bp deletion genotype completely knocks out production of BLG. In addition, we showed that the *LGB* knockout animals are free of any TALEN-mediated off-target mutations or vector integration events using an unbiased whole genome analysis. Our study demonstrates the feasibility of generating precisely biallelically edited cattle by zygote-mediated editing for the safe production of hypoallergenic milk.

## Introduction

BLG is a major allergen in cows’ milk with about 3% of babies and young infants affected by bovine milk allergies^1^ that can cause symptoms ranging from mild to live threatening^2^. Removal of BLG from cows’ milk reduces its allergenic potential and produces a milk that could provide a valuable source of nutrition for those children that currently cannot consume milk due to allergic reactions that are directed against BLG. A variety of processing technologies, including enzymatic hydrolysis, can be used to reduce the allergenicity of milk proteins but they are expensive and may lead to the appearance of new problematic epitopes^3^. Alternatively, genetic modifications designed to disrupt BLG production in animals have been trialed. Once established, such animals would be an attractive source for hypoallergenic, BLG-free milk. A relatively small number of animals might be sufficient to provide a niche product for commercial distribution given regulatory market-approval. Genome editing by targeting the BLG-encoding gene with zinc finger nucleases (ZFNs) followed by somatic cell nuclear transfer (SCNT) resulted in biallelically modified calves. Because the ZFN-cut yielded deletions that did not disrupt the reading frame, the animals were expected to still produce slightly shorter versions of the protein^4^. RNA interference utilizing microRNAs targeting the BLG-encoding transcript proved to be so efficient, that no BLG could be detected in a cow with a tandem microRNA transgene^5^. TALE nickase-assisted insertion of a human serum albumin cassette in the *LGB* locus resulted in the low frequency (0.1%) event of one biallelically targeted bovine fibroblast cell line which enabled the generation of cows whose milk lacks BLG^6^. The emphasis on this study, however, was on the high level expression of human serum albumin for further purification from milk and not at using the BLG-free, human serum albumin-rich milk as food. Similarly, BLG knockout combining TALENs-mediated gene editing and SCNT was also performed in goats and resulted in targeted mono- and bi-allelic gene exchange of BLG against either human lactoferrin (hLf) or human alpha-lactalbumin (hLA)^7,8^. Biallelically targeted BLG ^hLf/hLf^ goats were shown to no longer produce BLG but high levels of hLf ^8^, whereas monoallelically targeted BLG^+/hLA^ goats exhibited slightly reduced BLG and high expression of hLA^7^.

As outlined in the previous examples, the combination of gene editing and SCNT constitutes a powerful means to produce precisely targeted livestock animals. However, even 20 years after Dolly, SCNT is still hampered with poor efficiencies for the production of live offspring and losses after birth are also considerably higher in SCNT clones compared with sexually-derived animals. As an alternative to SCNT, zygote injections of genome editors have been successfully used to precisely change the genome of zebrafish, mice, rats, and rabbits^9–13^. In these fast-reproducing species with large numbers of offspring, non-mosaic homozygosity of the desired modification can be fairly quickly and cost-efficiently achieved by breeding from animals that are heterozygous and/or mosaic carriers of the genome edit. In livestock animals with longer generation intervals and typically smaller litter sizes, especially in the ruminant species, SCNT has been thus far the preferred route and zygote injection has only recently been trialed. Aiming to produce African swine fever virus resistant pigs, Lillico *et al*. introgressed a naturally-occurring allele from the African warthog into European-breed pigs by zygote injection of ZFNs and a plasmid DNA donor^14^. Out of the resulting 46 piglets, three were edited and one shown to be a non-mosaic, biallelically modified animal. In pigs, low efficiencies for the desired generation of homozygous gene edited animals can be overcome by large litter sizes whereas in goats, sheep or cattle with small litter sizes, direct embryo transfer would result in the costly and undesired generation of animals that are mostly not edited or heterozygous or mosaic for the genome edit. In order to restrict the transfer of embryos to those that are highly modified, we developed a new method combining zygote injections in bovine with biopsy pre-implantation diagnosis and embryo cryopreservation prior to transfer in recipients. We applied this new method for the generation of BLG knockout calves. Here, we describe the production of three calves, two male and one female that are biallelically edited for a precise *LGB* mutation and are essentially non-mosaic. This proves the ability of zygote-mediated genome editing to generate precision-edited cattle for the dissemination of valuable phenotypes. We further show the effectiveness of the nine bp deletion that prevented the production of BLG in a hormonally induced lactation of the female calf.

## Results

**Figure 1:**
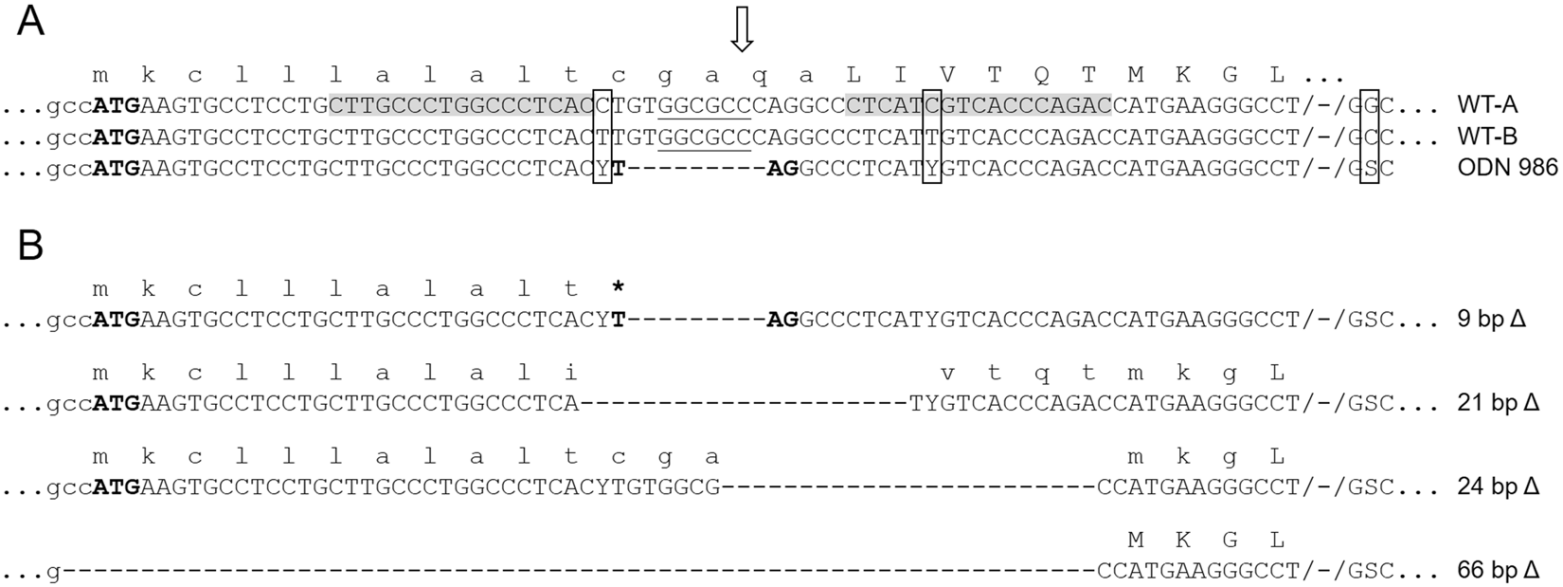
*LGB* target region, HDR template and identified edits in the produced calves and their corresponding embryos. (**A**) shown is the DNA sequence for the two main wild type *LGB* variants A (WT-A) and B (WT-B) with the TALEN binding sites (grey boxes) and the cleavage site (arrow). The sequence of the HDR template (ODN 986) is aligned and contains two Y and one S nucleotide at three polymorphic sites (boxed) between WT-A and WT-B. The underlined sequence indicates the recognition site of the restriction enzyme SfoI. Start and Stop codons are highlighted in bold. (**B**) shown are the aligned sequences of edited alleles showing the endpoints of the four deletions (9 bp Δ, 21 bp Δ, 24 bp Δ, 66 bp Δ) that were identified in this study. Deletions are represented as dashes. The corresponding amino acid sequences are given in single letter code above the DNA sequences with lower case indicating amino acids of the signal peptide or putative signal peptide (21 bp Δ, 24 bp Δ) and upper case amino acids of the mature protein.

We previously demonstrated that co-injection of a homology-directed repair (HDR) template and TALENs into bovine zygotes efficiently generated blastocysts with biallelic edits for a premature stop codon in the *LGB* gene (Fig. 1A)^15^. We now applied this same strategy to generate live *LGB* knockout calves.

### Generation and characterization of precisely edited embryos for transfer

Following the co-injection of the TALEN pair btBLG1.2 and ODN 986 as HDR template into zygotes produced by *in vitro* fertilization (IVF), embryos were cultured to the blastocyst stage (see Supplementary Table S1). To ensure that only appropriately edited embryos were transferred into recipient cows for development to term, we wanted to evaluate the extent of precise genome editing in individual embryos prior to their transfer. Hence, we sampled a small biopsy from the trophoblast of each embryo, ranging from 10 to 15 cells, which were used to extract genomic DNA for subsequent PCR analyses. Meanwhile, the biopsied embryos were cryopreserved and stored until the results became available for the transfer of embryos that were verified for the intended edit.

All embryo biopsies were first screened for the presence of the introduced nine bp deletion using a nested PCR strategy. Following amplification of the *LGB* target locus, a second PCR using an internal primer with binding specificity for the genome edited nine bp deletion allele identified embryos that were precisely edited^15^. Approximately 32% (75/234) of all biopsy samples were PCR-positive based on this assay (see Supplementary Table S1).

Next, the results from the nested PCR were verified by a TaqMan PCR assay using a hybridization probe that was designed to be specific for the nine bp deletion allele. Two thirds (50/75) of the biopsy samples that were positive by nested PCR, representing approximately 21% (50/234) of all samples, were confirmed to carry the desired nine bp *LGB* deletion by the TaqMan assay (see Supplementary Table S1). To estimate the abundance of genome edited alleles and potential degree of mosaicism in individual embryos, we calibrated the TaqMan results with the results of a quantitative PCR assay for the endogenous bovine gene *CSN2* as a reference gene. With this information, we scored and ranked 26 candidate embryos according to the abundance of genome edited alleles (Table 1). Five of the top-ranked embryos were further validated for a high degree of knockout alleles by subcloning the amplified fragment from the *LGB* target region and sequencing of individual subclones. Four of the five analyzed embryos exclusively yielded plasmid subclones with the nine bp deletion (with 5 to 14 subclones analyzed per embryo) while one embryo yielded 10 out of 11 modified subclones and one carrying the wild type allele (Table 1). Together, the analyses verified that embryos possessing predominantly the edited nine bp deletion allele were generated and identified for the subsequent transfer of suitable embryos to generate biallelic *LGB* knockout calves.

**Table 1:** Summary of the treatment and characterization of the edited biopsied and cryopreserved embryos prior to transfer.

Embryos				Biopsies				
ID	Grade	Cryo-preservation	Post thaw grade	Transfer	Nested PCR	TaqMan	Score	Sub-clones
TS91	XB1	slow frozen	XB1	yes	+ve	+ve	26.4	n/a
TS37	XB2	slow frozen	dg	no	+ve	+ve	18	n/a
TS144	B2/B1	slow frozen	B2	yes	+ve	+ve	17.5	n/a
TS121	XB1	slow frozen	dg	no	+ve	+ve	17.2	n/a
T49	XB2	vitrified	XB3	yes	+ve	+ve	17	n/a
T53	XB2	vitrified	XB2	yes	+ve	n/a	n/a	n/a
T35	B2	slow frozen	XB2	yes	+ve	+ve	16.3	9/9
TS147	XB2	slow frozen	dg	no	+ve	+ve	16.2	n/a
TS29	B2	slow frozen	B2	yes	+ve	+ve	16.1	6/6
T24	XB2	vitrified	XB3	yes	+ve	+ve	15.6	14/14
T46	B2	vitrified	dg	no	+ve	+ve	15.5	n/a
TS26	XB2	slow frozen	XB2	yes	+ve	+ve	14.8	n/a
TS70	eB2	slow frozen	dg	no	+ve	+ve	14.5	5/5
TS47	XB2	slow frozen	dg	no	+ve	+ve	14.3	n/a
T33	XB1	slow frozen	XB2	yes	+ve	+ve	13.4	n/a
T22	XB2	vitrified	dg	no	+ve	+ve	13	10/11
TS14	B2	slow frozen	B2	yes	+ve	+ve	12.8	n/a
TS23	B2	slow frozen	dg	no	+ve	+ve	12.2	n/a
TS36	XB2	slow frozen	XB3	yes	+ve	+ve	12.1	n/a
TS122	B2	slow frozen	B2	yes	+ve	+ve	12.1	n/a
T23	XB1	vitrified	XB2	yes	+ve	+ve	11.7	n/a
TS62	B1	slow frozen	B2	yes	+ve	+ve	11.7	n/a
TS125	XB2	slow frozen	lost	no	+ve	+ve	10.7	n/a
TS82	XB2	slow frozen	dg	no	+ve	+ve	10.3	n/a
TS139	B2	slow frozen	n/a	n/a	+ve	+ve	9.7	n/a
T90	B2	vitrified	n/a	n/a	+ve	+ve	9.1	n/a
TS126	B2	slow frozen	n/a	n/a	+ve	+ve	8.1	n/a

Note: B1 = grade 1 blastocyst; B2 = grade 2 blastocyst; XB1 = expanded blastocyst grade 1; XB2 = expanded blastocyst grade 2; eB2 = early blastocyst grade 2; +ve = positive; −ve = negative; dg = degenerate; lost = embryo lost during warming; n/a = not applicable.

### Generation of live BLG knockout cattle

**Figure 2:**
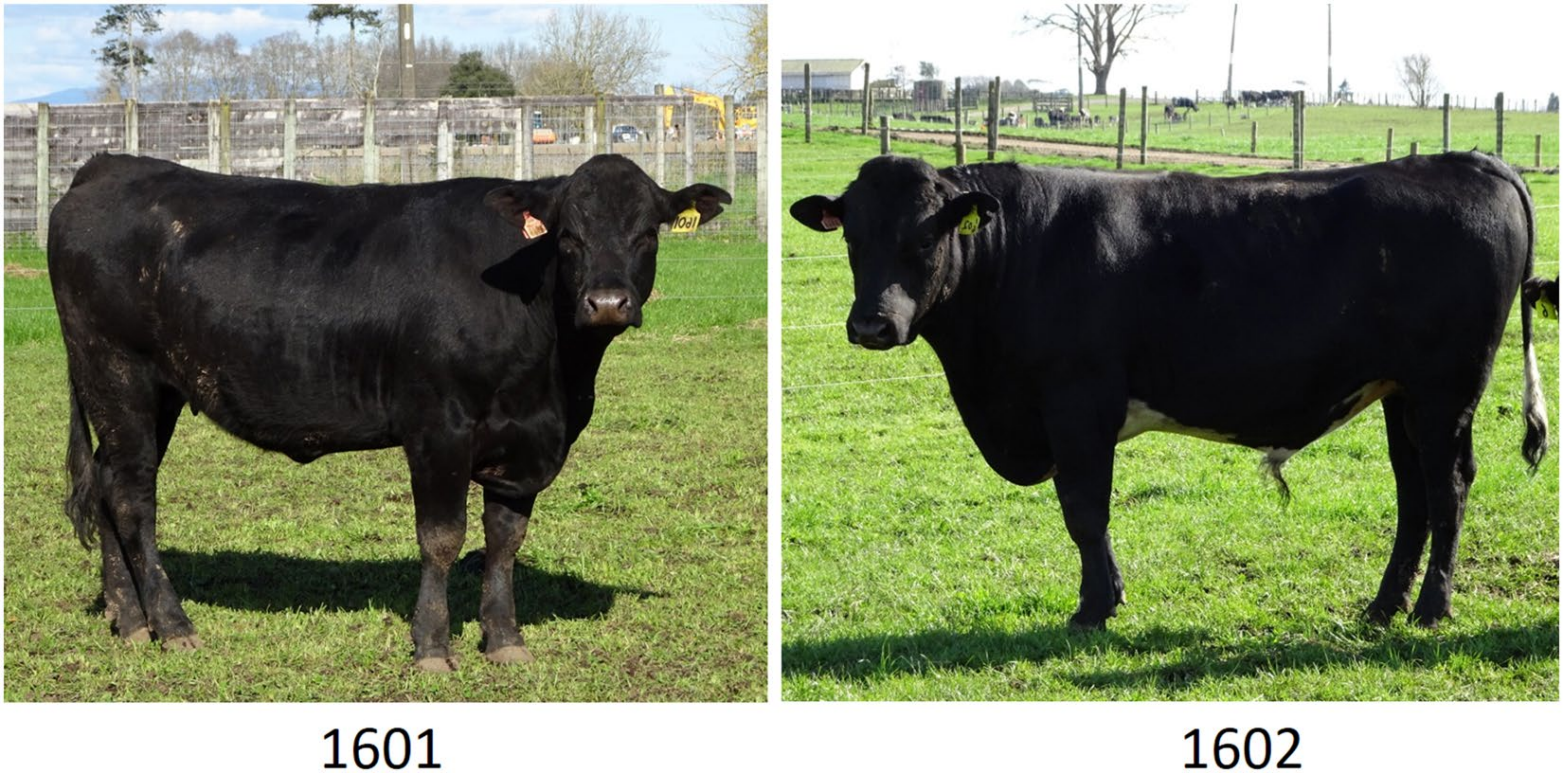
Cattle, genome-edited for a precise disruption of the *LGB* gene. Shown are the 1601 female and 1602 male with biallelic edits of a repair template-directed nine bp deletion at the age of 19 months that were produced by zygote-mediated HDR editing.

For the transfers, we selected a total of 24 high-ranking (score >10) cryopreserved embryos (Table 1). Following thawing, we observed ten degenerate-looking embryos which we no longer considered suitable for transfer and lost one embryo due to a broken straw (Table 1). The remaining 13 embryos were categorized as grade one to grade three embryos after thawing and transferred individually to recipients for development to term. A first ultrasound scan on day 35 of gestation identified three established pregnancies. All three pregnancies went to term. Two calves, a female calf (1601) and a bull calf (1602), were healthy and thriving (Fig. 2), while the third calf (424), a male, died shortly after birth.

### Characterization of the edited genotype

**Figure 3:**
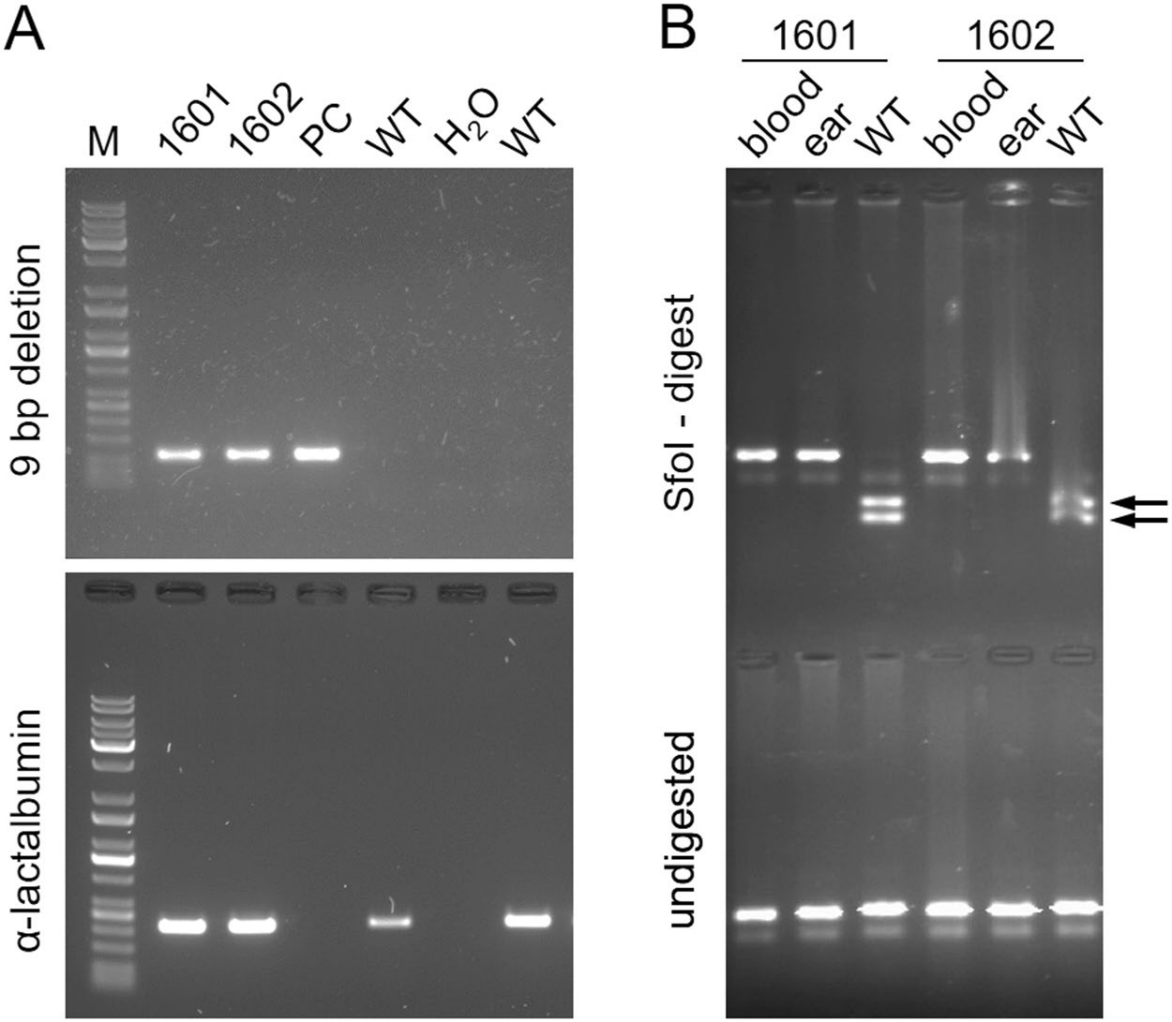
Detection of ODN-mediated genome edits by edit-specific PCR and restriction analysis. (**A**) Shown are amplification results for the nine bp deletion-specific PCR and a control amplicon for an endogenous gene (α-lactalbumin) with DNA isolated from a blood sample from the two live calves 1601 and 1602. PC: positive plasmid control with nine bp *LGB* deletion insert; WT: genomic wild type DNA; H_2_O: water; M: DNA size marker. (**B**) Results of a diagnostic restriction enzyme digest with SfoI on blood and ear derived DNA from calves 1601 and 1602 and a wild type DNA sample. The black arrows point to two diagnostic restriction fragments indicative of an unedited wild type sequence. The bottom gel shows the undigested PCR fragments. The gel images in panels A and B were cropped for improved clarity. The respective full size gel photos are shown in Supplementary Figure S3.

**Figure 4:**
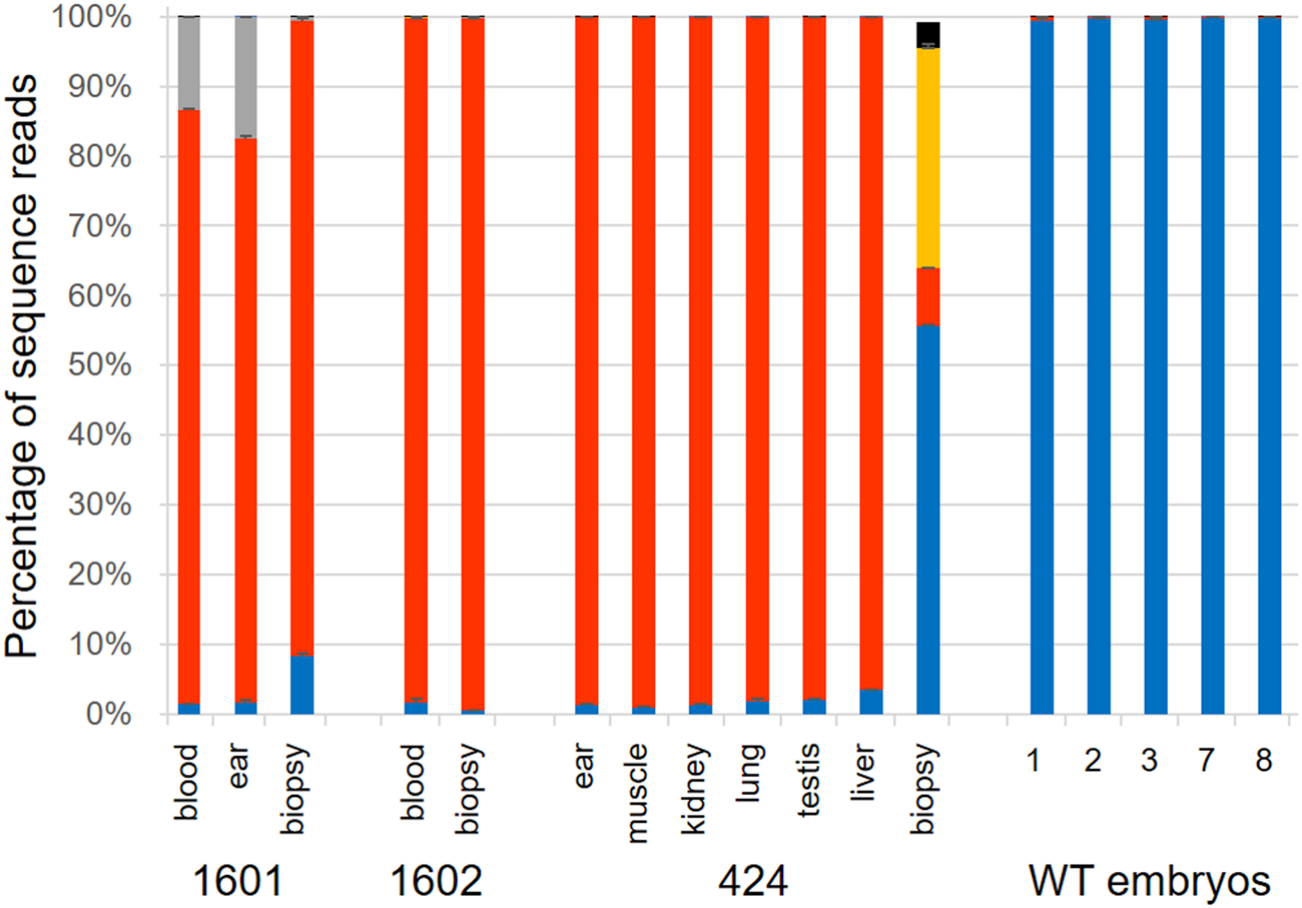
Deep sequencing analysis of the edited *LGB* locus in the calves 1601, 1602 and 424 and corresponding embryo biopsies. DNA isolated from blood, ear fibroblasts (ear), a selection of tissues (as indicated) and embryo biopsies (biopsy) were analyzed for the presence of TALEN-mediated edits and unaltered wild type sequences of the *LGB* target locus by enumerating corresponding sequencing reads for 1601, 1602, 424 associated samples in addition two five wild type embryo samples (1, 2, 3, 7, 8). The bars depict the percentage of all joined paired end sequence reads in the indicated samples for the HDR-generated nine bp deletion allele (red), wild type allele (blue) and additional 21 bp (grey), 24 bp (yellow) and 66 bp (black) deletion alleles that were identified by the deep sequencing analysis. The values that were used to generate the graph can be found in Supplementary Table S2. Error bars indicate standard errors.

Blood samples were taken to isolate DNA and genotype the two surviving calves with the mutation-specific PCR. Both calves showed the presence of the edited nine bp deletion (Fig. 3A). Next, we isolated fibroblasts from an ear punch as a different source of DNA to determine the potential degree of mosaicism in two different cell populations and whether the two animals are likely to be fully modified, non-mosaic *LGB* knockouts. Following amplification of the *LGB* target locus, the PCR product was digested with SfoI, a restriction site that is present in the wild type allele but deleted in the edited allele (Fig. 1). The *LGB* sequence derived from blood and ear fibroblasts remained completely undigested for both calves with no detectable cleavage products (Fig. 3B). This indicated a very efficient genome conversion to the edited genotype in these calves and no or very few remaining wild type alleles present. To more accurately quantify the presence of edited and wild type alleles and to assess the occurrence of the nine bp deletion versus non homologous end joining (NHEJ) repair-generated indels at the cleavage site, we determined the number of sequence reads for edited and wild type alleles following deep sequencing of the PCR-amplified *LGB* target locus in two independent sequencing runs (Fig. 4; Supplementary Table S2). For the female calf 1601, 85% and 81% of all sequence reads from blood and ear-derived DNA, respectively, were of the edited allele sequence with a nine bp deletion. However, we also detected a 21 bp deletion at a level of 13% and 17% in blood and ear fibroblasts, respectively, that extended three bp upstream and nine bp downstream of the intended nine bp deletion and caused an in-frame deletion of seven amino acids (Fig. 1B). The wild type allele was measured at 1% and 2%. Calf 1602 showed a high conversion to the nine bp deletion genotype for blood (98%) and only a low level of a residual presence of the wild type allele (2%). Due to a poor sequencing result for the ear fibroblast sample, producing less than 1300 joined, mapped sequence reads, no reliable quantification of the edited allele was possible for this tissue.

The non-surviving male calf (424) provided the opportunity to analyze a wider range of tissues. All analyzed tissues showed an almost exclusive presence of the nine bp deletion allele (ear 99%, muscle 99%, kidney 99%, lung 98%, testis 98%, liver 97%).

We then compared the results for the calves to the edited allele frequency in the corresponding biopsies from the blastocysts that later developed into the three calves (Fig. 4, Supplementary Table S2). The frequency of the nine bp deletion allele in the biopsy of embryo TS26 (which produced calf 1601) was determined to be 91%. The wild type allele was detected at 8% while the 21 bp deletion was only identified in one of the sequence runs with 1% of all paired sequence reads. For the biopsy of embryo TS62 (which produced calf 1602), the nine bp deletion had a contribution of 99%. Overall, editing in the biopsy samples was consistent with the results for calves 1601 and 1602. This was in stark contrast to the biopsy from embryo TS29 which produced the non-surviving calf 424. Despite the calf showing >97% contributions of the nine bp deletion allele in all tissues studied, only much lower levels (8%) were detected in the corresponding embryo biopsy sample. Instead, the majority of sequence reads represented the wild type allele (56%). In addition, we detected the presence of 24 bp (32%), and 66 bp (4%) deletions, overlapping the site of the nine bp deletion (Fig. 1B).

For wild type controls, we assessed sequence reads from non-injected embryos (WT1 – WT 3) generated with semen from the sire used for the production of calves 1601 and 424, and embryos WT7 and WT8 generated with semen used for the production of calf 1602. Sequence reads of the five wild type control embryos mainly comprised the sequences of the wild type allele (>99%) with the sample of embryo WT1 also showing low levels (1%) of sequence reads with the nine bp deletion, possibly indicating some minor contamination.

Because of the poor sequencing results for the 1602 ear sample and greatly different composition in the TS29 biopsy sample compared to the 424 tissue samples, we decided to also quantify the allelic composition in all our samples by digital droplet PCR (ddPCR, see Supplementary Fig. S1). For the calf 1602, we could complement the deep sequencing data and clarify the composition of the ear fibroblast sample. In these cells, the genotype was exclusively made up of the nine bp deletion allele (100%). In contrast to the deep sequencing result, the ddPCR result for the 424 embryo biopsy was consistent with the adult tissues, and also showed a high conversion rate (100%) into the nine bp deletion genotype. All other samples essentially mirrored the deep sequencing results including the presence of another allelic variant in the 1601 samples. Using tracking of indels by decomposition (TIDE) analysis^16^ on the sequence of the 1601 *LGB* target region PCR fragment in comparison to the equivalent wild type fragment, we could identify the other allelic variant as the 21 bp deletion allele. According to the TIDE analysis, the 21 bp deletion allele was present at a level of 9.7%, with the main nine bp deletion allele representing 89.1% (see Supplementary Fig. S2).

### Off-target analysis

To enable an unbiased assessment of potential off-target mutations, we sequenced the whole genome of the two live calves 1601 and 1602 with a 30-fold coverage. We compared the whole genome sequencing reads of the *LGB* knockout cattle with the bovine reference genome to identify sequence variations that were then correlated with the juxtaposition of degenerate binding sites for each of our TALEN monomers using PROGNOS^17^. This identified a total of 316 potential off-target sites considering hetero- and homodimeric TALEN pairs. The distributions of these sites across the different degeneracy levels are summarized in Table 2. Examination of the sequence within 20 bp on either side of predicted potential targets from relevant whole genome reads derived from the *LGB* knockout cattle failed to detect any TALEN-induced off-target events. The single variant identified by the analysis was the intended on-target editing event and thus, both animals are devoid of any off-target modifications. It should be noted that despite the sequencing of the whole genome with 30-fold coverage, the on-target event exclusively comprised the nine bp deletion edit and no sequence reads with a 21 bp deletion were detected in 1601 and 1602.

**Table 2:** Comparison of degenerate TALEN sites as potential off-target sites in the bovine genome to variants called from whole genome shotgun sequencing reads.

Degeneracy in a TALEN pair	Genome-wide off-target sites	Variants from whole genome shotgun sequencing reads^*^	
		1601	1602
0_0	1	1	1
1_4	0	0	0
1_5	0	0	0
4_2	1	0	0
3_3	0	0	0
2_5	3	0	0
3_4	2	0	0
3_5	14	0	0
4_4	6	0	0
5_4	60	0	0
5_5	229	0	0
Total	316	1	1

*Within 20 bp of a degenerate TALEN sites.

The whole genome sequence information was also mapped against the sequence of the TALEN vector. Only 239 and 266 out of 332,493,118 and 341,773,863 read pairs for samples 1601 and 1602, respectively, had one or both reads mapping to the vector with all of the mappings located in only the Xenopus beta-globin 3′ UTR region of the vector. The corresponding pairs mapped to locations scattered across the genome of the *LGB* knockout cattle indicating spurious hits with regions of sequence homology between the vector and the genome. This provided strong evidence that the plasmid vectors, used to deliver the *LGB*-specific TALENs were only transiently present and did not integrate into the genome of the edited cattle.

### Milk phenotype of the female *LGB* knockout calf

**Figure 5:**
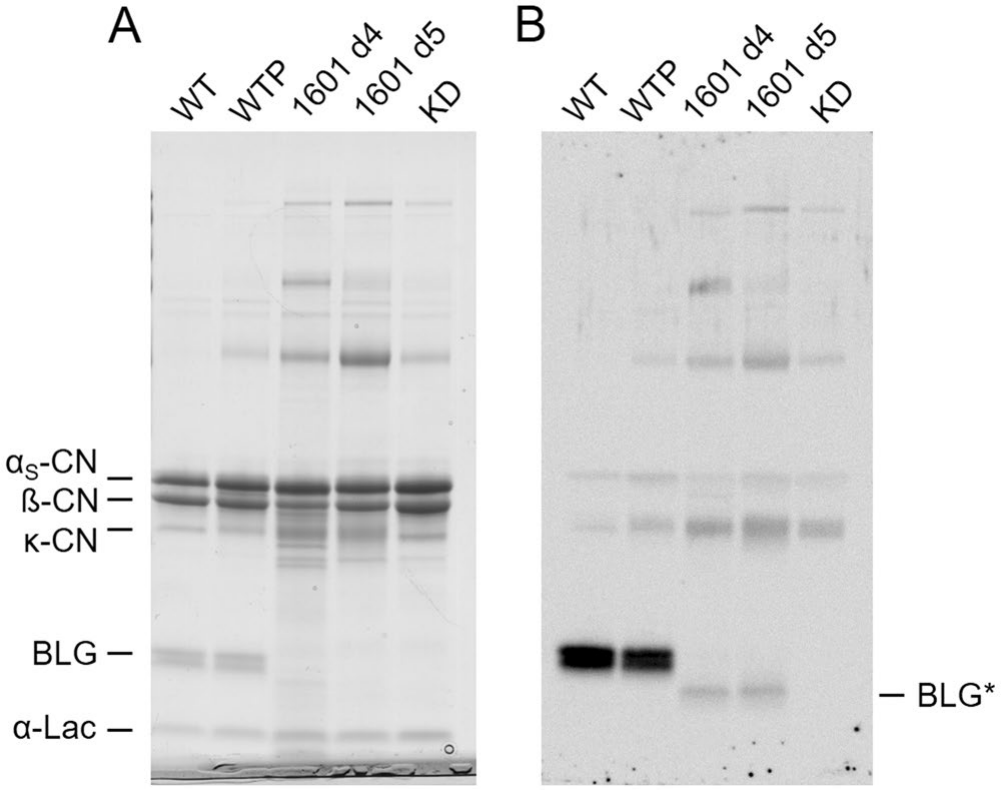
Amount of BLG in induced milk of the genome edited *LGB* knockout calf 1601. BLG levels in the milk of calf 1601 were assessed by (**A**) Coomassie Blue staining and (**B**) western analysis following SDS-PAGE separation of milk samples. Each lane of the gel was loaded with an equal amount of milk. WT: natural milk from a single wild type cow; WTP: pooled natural milk samples from several wild type cows; 1601 d4 and d5: milk sample from 1601 on the fourth and fifth day of an induced lactation; KD: milk sample from a transgenic line of *LGB* knockdown cattle. Positions of the main milk proteins and a smaller form of BLG (BLG*) observed by western are indicated. α_s_-CN: α_s_-casein; β-CN: β-casein; κ-CN: κ-casein; BLG: β-lactoglobulin; α-Lac: α-lactalbumin. Please note, the images shown in panels A and B were cropped for improved clarity. The respective full size photos of the stained gel and western membrane are shown in Supplementary Figure S4.

The female calf 1601 was hormonally induced into lactation at eight months of age. The major milk components were determined by Fourier Transform Infrared analysis. The milk produced by calf 1601 on day 5 of lactation showed lower values for lactose and was higher in protein compared to wild type induced and natural milk samples (see Supplementary Table S3). In addition, the 1601 milk had slightly higher levels of nonfat solids. A control sample from a transgenic BLG knockdown cattle line^5^ showed a similar trend with the exception of lactose which was similar to the wild type controls. For a more detailed assessment of the milk protein composition of the 1601 sample, milk proteins were separated by SDS gel electrophoresis and analyzed following Coomassie Blue staining and western detection of BLG (Fig. 5). The Coomassie staining visualized all major milk proteins in the wild type milks but failed to detect BLG in the samples from 1601 and the control sample for BLG-free milk of the transgenic knockdown line.

Relative quantification of αS-casein, β-casein and α-lactalbumin in the 1601 and knockdown milk samples compared to the pooled wild type sample showed increased levels for αS-casein (134% and 163%) and α-lactalbumin (143% and 182%) in 1601 and the knockdown milk over the control sample. While β-casein was also higher (157%) in the knockdown sample it was lower in the sample from calf 1601 (86%).

Detection by western analysis with a bovine BLG-specific antibody showed a strong BLG-specific signal in wild type milk samples. The corresponding protein was absent in the milk of the genome edited calf and the knockdown control sample. However, exclusively in the 1601 samples, the antibody recognized a smaller protein (BLG*) that was present at relatively low levels compared to mature BLG observed in the wild type control samples (Fig. 5).

**Figure 6:**
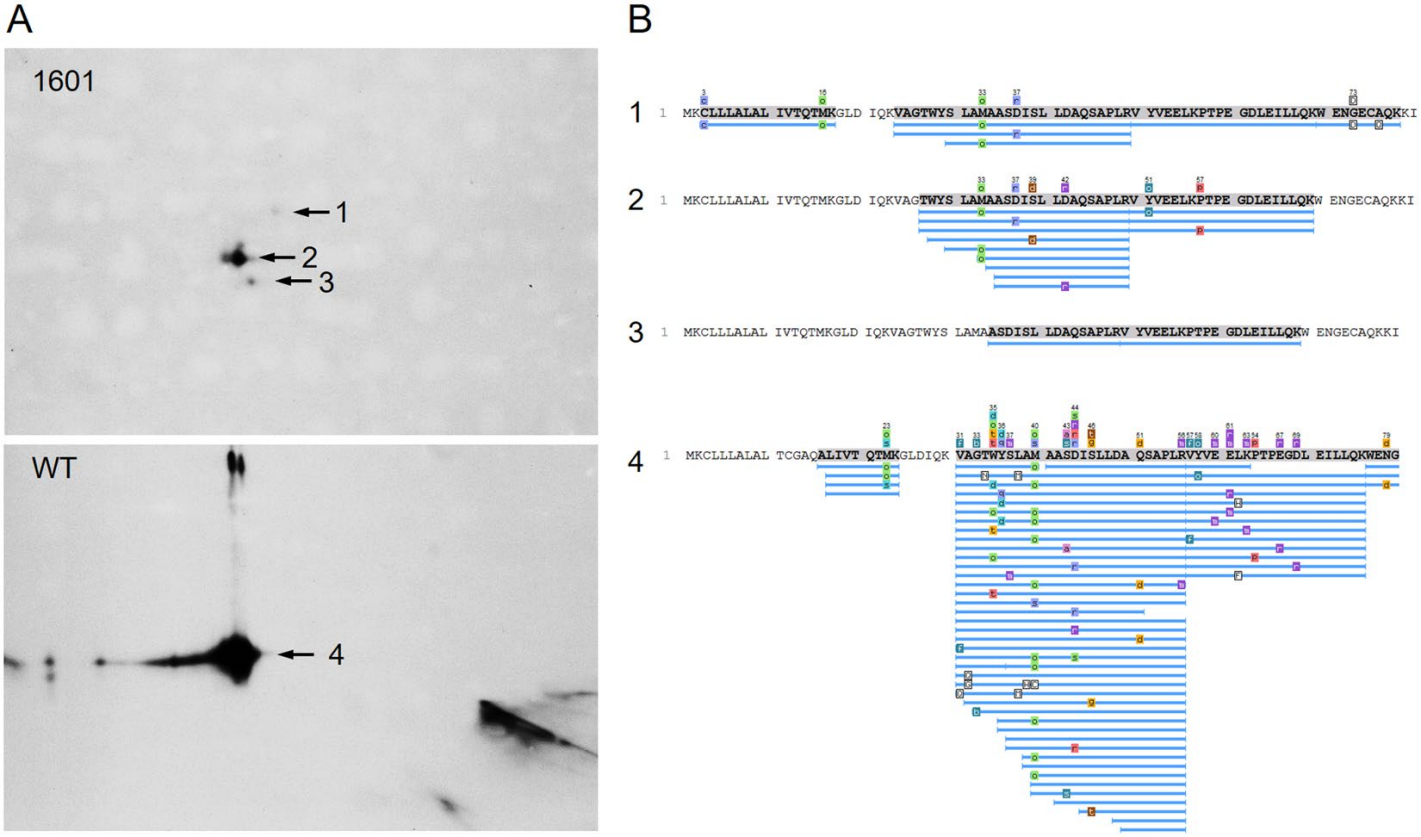
Identification of the smaller, immunoreactive BLG variant. (**A**) Shown are western blots of the milk protein following 2D separation performed with 1601 and wild type milk samples. The images of the stained western membranes were cropped for improved clarity. The respective full size photos of these membranes are shown in Supplementary Figure S5. (**B**) Peptides (blue bars), identified by mass spectrometry associated with the protein spots corresponding to the BLG-specific signals 1, 2, and 3 (1601) and 4 (WT). Bold letters indicate amino acids represented in detected peptides and colored boxes denote amino acids with different post-translational modifications.

To determine the identity of this smaller protein, the milk proteins were first separated by two-dimensional gel electrophoresis (2-DE). Using western detection with a BLG-specific antibody, we detected one major immunoreactive protein spot (spot 2) and two additional spots (spot 1 and 3) with comparatively low signal intensities (Fig. 6). Together with the spot for wild type BLG (spot 4), we analyzed these three spots by mass spectrometry. All spots were identified as derivatives of BLG. The most N-terminal peptide detected for spot 1 had an amino acid sequence consistent with the loss of seven amino acids caused by the 21 bp deletion allele we detected in calf 1601. This confirmed the expression of the unprocessed form of this new variant form of BLG. For the major spot 2, this N-terminal peptide was not detected. Here, the most N-terminal peptide started at position 27 (TWY). According to signal peptide predictions, the modified amino acid sequence in this variant constituted a signal peptide with cleavage between amino acids 18 and 19 (MKG-LDI). Hence, spot 2 most likely represents the processed form of the seven amino acid deletion variant. The detected N-terminal peptides for spot 3 started another five amino acids further towards the C-terminal end (amino acid 32, ASD) indicating that the smallest immunoreactive protein spot has undergone some proteolytic processing with a further truncation of the N-terminal end. The main protein spot for wild type BLG (spot 4) produced peptides was consistent with the mature protein and a processed N-terminus starting at amino acid 17 (AQA-LIV).

## Discussion

For sufferers of cows’ milk allergy, milk from other mammals such as sheep and goats have been considered as an alternative milk source. However, these hold significant risks because of the potential for cross-reactivity. Instead, a diet excluding the causative allergens provides the most efficient treatment option^18^. The precision and efficiency of genome editing makes it possible to completely eliminate specific allergens from cows’ milk through disruption of the reading frame of the corresponding gene, or where known, the antigenic epitope. This provides a novel avenue to generate cows that produce a milk with hypoallergenic properties.

Disruption of the *LGB* gene which encodes BLG, a major allergenic protein in dairy milk, has been approached by the introduction of site-specific indels. These can be very efficiently generated following NHEJ repair triggered by a double strand break that is introduced with a programmable nuclease^4,19^. While such undefined mutations are very efficient in generating frame shift mutations and disrupting reading frames, they pose the risk of generating entirely new strings of amino acids and potentially new allergenic epitopes encoded by the novel reading frame. To avoid this risk, we have introduced a precise modification by HDR that disrupts the *LGB* gene by creating a premature stop codon in the original reading frame. Although the mutation is engineered it mimics a natural mutation with the deletion of only a small DNA segment. This or similar mutations might already exist in the natural population but without substantial efforts aimed at identifying such mutations, they are unlikely to be found.

Compared to NHEJ editing, the HDR approach is less efficient. For zygote-mediated editing, chosen for its high efficiency to generate live animals, a low HDR editing efficiency can easily translate into the generation of unwanted non-edited, NHEJ-edited or highly mosaic animals carrying a significant risk of animal wastage^14,19^.

We have previously shown that it is possible to generate embryos with a high conversion rate to a HDR-generated, nine bp *LGB* deletion allele^15^. To minimize animal usage for the production of cattle with a precise knockout of the *LGB* gene, we have biopsied and pre-screened injected embryos enabling us to only transfer validated embryos. For logistical reasons, the biopsied embryos needed to be cryopreserved to analyze and accumulate suitably edited embryos. In the present study, the cryopreserved embryos recovered poorly and resulted in an overall low development rate to term of 23%. In another trial with cryopreserved biopsied cattle embryos, >50% of the transferred embryos developed into live calves (GL, unpublished results). This indicates that biopsied and cryopreserved embryos do not generally show low developmental competency but that the problems arose most likely due to technical issues with the cryopreservation of these particular embryos.

All three calves showed a high degree of conversion to the edited nine bp knockout genotype in cells from all tissues that were analyzed. Two male calves showed a biallelic HDR knockout, non-mosaic genotype. In the female calf 1601, we identified in addition to the major HDR knockout allele another, most likely NHEJ-generated on-target deletion of 21 bp, representing approximately 15% of all alleles. There was also a good correlation between the genotypes determined for two of the embryo biopsies and their respective calves. By contrast, the deep sequencing results for the biopsy of the third embryo was very different to the results with six different tissues isolated from the corresponding male calf 424. Re-analysis of the original biopsy sample by ddPCR confirmed a genotype that mirrored the genotype of the calf. Considering the inconsistency of the biopsy sequence results with the genotype of the calf, it most likely implies that the sequenced sample might have been from a different embryo or the discrepancy arose from an artifact generated during amplification, library generation or sequencing. Overall, the characterization of the calves demonstrated that the strategy of pre-screening edited embryos worked and that it is feasible to produce highly HDR-edited, and non-mosaic cattle using the microinjection approach. The bull calf 1602 was confirmed to be fully converted to the nine bp *LGB* deletion genotype and represents a true founder for this novel genotype. Although HDR-edited for the nine bp disruption allele to a high degree, the female calf 1601 also had a minor allele responsible for producing small amounts of a shorter BLG variant. This shorter variant only lacks a few N-terminal amino acids compared to the 162 amino acids for the wild type form of BLG and comprises the main suspected immunoreactive epitopes^20^. Thus, the observed shorter BLG variant is likely to still cause an allergic reaction. However, the minor allele and production of the shorter variant can be easily eliminated by segregation of the alleles in the next generation.

Taking into account that up to 46% (Table S1 and ref.^15^) of the injected blastocysts were positive for the HDR-edited allele and only approximately 10% were edited to a suitably high degree^15^, transfer of all injected blastocysts would have produced many unwanted calves. By contrast, the biopsy strategy and transfer of only validated embryos more likely ensures that all calves produced have the intended genetics. In addition, this equates to a 90% reduction in the number of recipient animals required. Based on a 10% efficiency for suitably edited embryos, the biopsy strategy would also favorably compare to the SCNT approach. Here, the use of a validated donor cell clone would only generate fully HDR-edited animals. However, the typically low efficiency for development to term (3–10%) could necessitate the use of up to a tenfold higher number of recipient cows compared to the transfer of injected and validated embryos.

Strictly speaking, we have only demonstrated the feasibility of our strategy for TALENs. One might assume that the conclusion can be readily extended to other programmable nuclease systems, such as ZFNs, and CRISPR/Cas9, all sharing the functional principle of introducing a double strand break at site-specific target sites. However, these are different systems and whether they differ in their performance characteristics is presently not well understood. A recent comparative study of TALENS and CRISPR/Cas9 provided some evidence in support of that argument and showed that TALENs might have a greater efficiency in generating HDR edits than CRISPR/Cas9^21^.

The main risk associated with genome editing is the unintended introduction of mutations at non-target sites due to the potential binding of a programmable nuclease to unrelated binding sites sharing some homology with the genuine target site. Although initial studies in human cancer cell lines reported high Cas9 off-target activities^22,23^, it appears that genome editing applications in non-transformed cells and mice have a low probability for off-target modifications^24–26^. This was also confirmed for genome edited livestock where only a few studies, and inclusive of multiplexed editing, reported on the detection of rare off-target mutations^27,28^. The great majority of studies that assessed off-target mutations described the intended on-target editing as the only genome editing-induced changes to the genome^29–37^.

Nevertheless, the potential exists and while continuous improvements have been made to increase the specificity of the different editing systems^38^, potential off-target events need to be considered.

In our study, we used whole genome sequencing for an unbiased genome-wide assessment which did not detect any off-target events in both of our live animals. However, the sequencing was performed at 30 fold coverage of the genome, which will limit the possible sensitivity. Because we were unable to detect the 21 bp on-target deletion representing <17% of all *LGB* alleles, we cannot entirely exclude the possibility for the presence of some low level off-target mutations in these animals.

The analysis of the induced milk demonstrated that the full length, mature BLG is no longer produced by 1601. Instead, we saw low level production of a smaller protein that was recognized by the BLG antibody. The low level presence of a 21 bp deletion at the *LGB* target site in this animal encodes a BLG variant that is lacking amino acids 11 to 17, with six of the amino acids located in the signal peptide of BLG. This new N-terminal sequence appears to still function as a signal peptide albeit with a different cleavage site. Using mass spectrometry, we were able to identify a low abundant protein spot as the unprocessed protein and the major immunoreactive spot as the processed protein. This strongly suggests that the smaller BLG-related protein observed at low levels in the milk is expressed from the small proportion of 21 bp deletion alleles we detected in calf 1601.

Characterization of the general milk composition indicated a slight increase in protein and lower lactose content. However, the analysis had to be based on a milk sample from a single animal comparing milk samples from induced and natural lactation and cows of various ages. Fat and lactose levels are still within the normal range of milk from New Zealand cows^39^. Whether an increased protein concentration is associated with the knockout genotype or rather the induced lactation will need to be evaluated by comparing natural lactation samples from several knockout cows.

The milk of the female 1601 lacked the mature BLG. Instead, we could only detect a BLG variant that was smaller in size and present at much lower levels than the mature BLG in wild type milk. We could show that this variant was produced from a 21 bp deletion allele that comprised 17% of all *LGB* alleles in the female calf 1601. By contrast, the main nine bp deletion allele resulted in a clean disruption of the *LGB* locus that was no longer capable of producing BLG.

Together, our study demonstrated the feasibility of generating precisely biallelically edited, essentially non-mosaic cattle, free of off-target mutations, by zygotic co-injection of TALENs and an HDR-template. The small nine bp deletion we edited into the *LGB* gene generated a premature in-frame stop codon within the signal peptide. Theoretically, such a mutation could also arise naturally, and because the stop codon is in-frame, it does not lead to any novel amino acid sequence as with out of frame deletions. This strategy minimizes the potential residual risk due to the application of genome editing. Thus, these cattle, edited for the ability to produce BLG-free milk should provide a valuable line of cattle for the safe production of hypoallergenic cows’ milk.

## Materials and Methods

All experiments were performed in accordance with the relevant guidelines and regulations and approved by New Zealand’s Environmental Protection Authority.

### TALENs pair *Bos taurus* btBLG1.2

The expression vectors for TALEN pair *Bos taurus* btBLG1.2 was constructed to target a region immediately downstream of the start codon of the bovine beta-lactoglobulin-encoding *LGB* gene as previously described^15^.

### ***In vitro*** embryo production, cytoplasmic zygote injection and embryo culture

The production, injection and culture of embryos to the blastocyst stage was essentially carried out as previously described^15^. Briefly, slaughterhouse-derived oocytes were fertilized with semen from one of three bulls used over the duration of the project. Following the removal of the cumulus cells, zygotes were co-injected with btBLG1.2 plasmid DNA (20 ng/µl) and ODN 986 (100 ng/µl) 18 h post fertilization and co-cultured in groups of ten embryos until embryonic day seven or eight.

### Blastocyst biopsy and cryopreservation of embryos

Grade 1 and 2^40^ bovine *in vitro*-produced blastocysts at embryonic days seven or eight of development were washed in protein-free Embryo Hold medium (AgResearch, NZ) supplemented with 0.1 mg/mL polyvinyl alcohol (PVA). A single embryo was placed into a 50 µL drop of the protein-free Embryo Hold medium at 38.5 °C. Using an ultra-sharp splitting blade (AB Technologies, NSW, Australia), 10–15 cells were cut off the trophoblast under 200x magnification. Two microliters of fetal calf serum (batch 76827101, Moregate, New Zealand) was then slowly added to release the embryo and the biopsy from their electrostatic adherence to the plastic surface of the Petri dish (Falcon, Becton Dickinson, NJ). The biopsy was rinsed in phosphate-buffered saline plus PVA and transferred in less than 1 µL to a PCR tube containing 2.5 µL sterile distilled water for subsequent genotyping. After each biopsy, the splitting blade was sequentially cleaned with 0.25% trypsin EDTA (Invitrogen, New Zealand), 75% ethanol and protein-free Embryo Hold medium to exclude any cross-contamination of individual embryo samples.

The biopsied embryos were each transferred into a fresh culture drop of Embryo Hold medium with 8 mg/mL bovine serum albumin (BSA; MP Biomedicals, New Zealand), overlaid with mineral oil and held at 38.5 °C for 2–3 h until cryopreservation.

Biopsied embryos were cryopreserved with two different methods. With the first method, biopsied embryos were slow frozen in Embryo Hold medium containing 20% FCS, 1.5 M ethylene glycol and 0.1 M sucrose. After 10 min equilibration at room temperature, embryos were individually loaded and sealed in 0.25 mL straws (Minitube, Germany). The straws were then placed vertically in a cryo-chamber (CryoLogic, Australia) at −6 °C for 10 min, with ice nucleation initiated after the first 2–3 min. Straws were then further cooled to −30 °C at a rate of −0.5 °C/min, maintained at −30 °C for 5 min and directly plunged into liquid nitrogen for long-term storage.

Alternatively, biopsied embryos were vitrified using the CryoLogic Vitrification Method (CVM; cryologic.com/cvm). Embryos were first incubated at 38.5 °C in Embryo Hold medium containing 20% FCS and 7.5% (v/v) each of ethylene glycol and dimethyl sulfoxide (DMSO) (VSI solution) for 2 min. This was followed by a second equilibration in Embryo Hold medium containing 20% FCS, 0.1 mM Ficoll 70, 1 M sucrose and 15% (v/v) each of ethylene glycol and DMSO (VSII solution) for 30 sec. The embryo was then transferred in 2.5 µL of VSII onto the nylon hook of a CVM fibreplug. Within 30–60 sec of the first exposure of the embryo to the VSII solution, the nylon hook containing the droplet with the embryo was placed onto the surface of a stainless steel cooling block equilibrated in a bath of liquid nitrogen. Following embryo vitrification, the fibreplug was then placed in a cooled sleeve and directly plunged into liquid nitrogen for long-term storage.

### Embryo thawing

Slow frozen embryos were thawed by firstly exposing the straws to the air for 5 sec after removal from liquid nitrogen, before being placed in a 20–25 °C water bath until the ice melted. After cutting the ends of each straw, embryos were expelled into Embryo Hold medium containing BSA and 0.25 M sucrose for 5 min and maintained in complete Embryo Hold for 2–3 h before assessment and transfer to recipient cows.

Vitrified embryos were warmed in four steps, in base medium comprising Embryo Hold with 20% FCS at 38.5 °C. Firstly, the fibreplug was rapidly removed from liquid nitrogen, the sleeve removed and the hook immersed in warming solution with 0.27 M sucrose. After 2 min, the embryo was transferred to medium containing 0.16 M sucrose for 3 min, followed by two washes in serum-enriched Embryo Hold for 2.5 min each. Embryos were incubated for 2–3 h before morphological evaluation and embryo transfer.

### Analysis of embryo biopsy samples

Trophoblast biopsy samples were whole genome amplified using Ilustra’s Ready-to-go GenomiPhi V3 DNA amplification kit. Precise ODN-mediated mutations in amplified samples were detected by nested PCR with the first PCR amplifying a PCR product of about 550 bp (primers 840 and 841, 60 °C annealing) followed by a second, mutation-specific PCR (primers 994 and 841, 68 °C annealing) using the 550 bp first PCR fragment as template. Sequences of all primers used in this study are listed in Supplementary Table S4. To confirm the presence of the ODN-mediated mutations in selected embryos, 550 bp first PCR amplicons were subcloned into pGEM Teasy and subjected to a TaqMan PCR assay (described below) and/or analytical restriction digests with SfoI.

To confirm the presence of the nine bp deletion in the biopsy samples and to quantitatively analyze them, TaqMan assays were performed using Quanta’s Accustart mix (Corbett RG 6000: 95 °C 3 min followed by 40 × 95 °C 10 sec and 62 °C 25 sec, *LGB* amplification primers 1051 and 1052 and 1048 as TaqMan probe (Biosearch Technologies)). To determine a score for the level of HDR editing in the embryo biopsies, we also quantified *CSN2* as an endogenous single copy gene, by quantitative PCR using Takara’s SYBR Premium mix (Corbett RG 6000: 95 °C 3 min followed by 40 × 95 °C 10 sec and 62 °C 25 sec, amplification primers 290 and 291). The score indicating the degree to which individual embryos were gene edited with the nine bp deletion was calculated by subtracting take-off values obtained for the TaqMan assays from take-off values obtained for the quantitative PCR of *CSN2*.

To analyze subcloned PCR fragments, part of the transformed bacterial colonies were directly transferred as template into the PCR reaction mixture of TaqMan assays.

### Embryo transfer

Following grading^40^, suitable quality embryos were non-surgically transferred to synchronized recipient cows as described previously^15,41^.

### Sequence analysis

Conventional sequencing of PCR fragments or subclones was carried out by Massey Genome Service (Palmerston North, New Zealand).

Embryo biopsies were whole genome amplified as described, while DNA isolated from blood and calf tissues were used directly as template DNA for PCR. Amplification with primers 1016 and 1017 then generated fragments that were compatible with the Illumina two step amplicon library preparation method. The next generation sequencing and evaluation of results were carried out essentially as described in our previous study^15^. Briefly, samples were sequenced in duplicate on an Illumina Miseq platform resulting in a total of 8,595,712 pairs of 250 bp paired end reads that gave 18,000 to 105,000 paired reads per sample with the exception of 3 samples: both replicates for the 1602 calf, ear fibroblast sample and one replicate for the wild type control embryo 8 sample which each had around 1,000 to 3,000 reads. Results from these three, low read count samples were omitted from the analysis. The reads were trimmed and joined as before^15^ and mapped to the target region of the bovine genome using BWA^42^ version 0.7.9a-r786 with default settings. Mutations were enumerated using the functions in Samtools^43^ version 1.1.

### ddPCR

All samples were assayed according to the method detailed in the Bio-Rad Droplet Digital™ PCR Applications Guide. Briefly, ddPCR reactions were prepared to a final concentration of 1 × Bio-Rad ddPCR Supermix for Probes (no dUTP), 900 nM primers, 250 nM target probe (FAM), 250 nM reference probe (HEX) and 750 nM dark probe (for reactions with HDR probe only), and either 100 ng of genomic DNA or 2 µl of 10 cycles preamplified PCR product from whole genome amplified (GenomiPhi, Ilustra) biopsy DNA. Droplets were generated with the QX100 Droplet Generator, transferred into a 96-well PCR plate and PCR amplified using a Bio-Rad PCR machine as follows: 95 °C for 10 min, followed by 40 cycles of 94 °C 30 sec, 60 °C 1 min (ramp rate 2 °C/sec), then 98 °C for 10 min. Samples were analyzed for HDR events using the HDR probe and for non-edited versus edited alleles with a drop-off probe. The data was acquired by a Bio-Rad Droplet Reader and analyzed using QuantaSoft™ Analysis Pro Software (Bio-Rad).

### TIDE

TIDE analysis has been designed to analyze CRISPR introduced indels^16^. To verify and quantify the predominant insertions and deletions introduced by TALEN editing in our samples, we entered a hypothetical sgRNA with a potential PAM sequence at the 3′ end of the introduced nine bp deletion in the TIDE calculator. We amplified the *LGB* target locus by standard PCR using primers 840/1015. The resulting 443 bp product (for the wild type allele) was sequenced and the resulting chromatogram sequence files compared to a wild type sequence to identify insertions or deletions.

### Signal peptide prediction

The possible presence and location of a signal peptide in the BLG variant encoded by *LGB* deletion alleles was determined using the SignalP 4.1 Server^44^.

### Off target and vector integration analyses

Two of the samples, 1601 and 1602, were sequenced using whole genome shotgun sequencing, producing 931,498,618 and 959,133,690 raw reads respectively. The raw reads were then trimmed with QuadTrim version 2.0.1^45^ using the default settings with the exception of raising the minimum base cutoff quality parameter from 15 to 20, resulting in 835,749,061 and 870,407,901 trimmed reads. The remaining reads were mapped against the *Bos taurus* UMD3.1 genome using the “mem” algorithm of BWA^42^ version 0.7.9a-r786 with default settings. Variants were called using GATK IndelRealigner^46^ version 2.3–9-gdcdccbb, resulting in a Variant Call Format (VCF) file. We then applied an iterative string search^17^ using the reference bovine genome build UMD3.1 to compute all potential off-target edits likely caused by the TALENs pair. Parameters for spacer allowance, mismatches and dimerization were set according to a previously described protocol^29^. All identified sequences were compared with the VCF files produced by the whole genome shotgun sequencing. A Perl script was used to find indels within 20 bp distance of predicted potential targets for the edited animals.

To determine potential vector integration, the trimmed whole genome shotgun reads were then mapped against the Vector sequence concatenated to the *Bos taurus* UMD3.1 genome using BWA^42^ version 0.7.9a-r786 with default settings. Samtools^43^ version 1.1 was used to extract read pairs where one of the reads mapped to the vector sequence with a mapping quality of greater than 10.

### Milk and milk protein analyses

The edited calf 1601 and a control calf were hormonally induced into lactation at eight months of age as previously described^5^. Wild type milk samples of natural lactation milk were collected from New Zealand dairy cows. Raw, whole milk samples were analyzed for the percentage of fat, protein, lactose, total solids and non fat solids using the Fourier transform infrared analyzer MilkoScan^TM^ Minor 6 (Foss Analytical, Hillerød, Denmark) calibrated to New Zealand bovine milk. Milk samples (0.1 µL) were analyzed by SDS gel electrophoresis with 8–16% Criterion™ TGX precast polyacrylamide gels (Bio-Rad) and the separated milk proteins stained with Coomassie Blue R 250. Signal intensities were quantified by Quantity One software (Bio-Rad).

Two-dimensional separation of milk proteins was performed essentially as described in our earlier studies^41,47^ and western detection of BLG was carried out according to published methods^5^.

### Mass spectrometry analysis

Positions of the BLG immunoreactive spots identified by western detection were located on the images of the Ponceau-stained membrane and corresponding spots on Coomassie-stained 2-DE gels were excised for mass spectrometry analysis. Gel pieces, approximately 1 mm^2^ in size, were excised for each selected 2-DE spot and processed for mass spectrometry as previously described^48^. Briefly, the Coomassie stain was removed from each gel piece, the proteins in the gel were digested with trypsin and the tryptic peptides extracted and lyophilized. The dried peptide extracts were reconstituted in 50 μL of 2% (v/v) acetonitrile in 0.2% (v/v) formic acid for subsequent mass spectrometry analysis essentially as previously described^49^. Briefly, peptides from each protein spot digest were separated and detected using a nano-Advance UHPLC (Bruker-Daltonics, Bremen, Germany) coupled to a maXis Impact ultra-high resolution quadrupole time-of-flight mass spectrometer (Bruker-Daltonics). For each sample, 5 μL was loaded onto a Magic C18AQ nano trap column (Bruker-Michrom, Bremen, Germany) in mobile phase buffer A, which comprised 0.1% (v/v) formic acid in water, at a flow rate of 5000 nL/min for 5 min. The trap column was switched in-line with an Intensity C18P analytical column (Bruker-Michrom) heated to 50 °C. Peptides were eluted from the trap column to the analytical column using a gradient of mobile phase buffer A and buffer B (0.1% (v/v) formic acid in 100% acetonitrile) increasing from 2% B to 45% B over 25 min at a flow rate of 800 nL/min. The eluate from the analytical column was directed to the captive spray ionization source (Bruker-Daltonics), and mass spectra were generated using data-dependent analysis mode with dynamic exclusion set to 0.2 min. The three most abundant precursor ions were automatically chosen for fragmentation during each MS scan in the mass range of 300–1250 m/z.

Peptide identification was performed using PEAKS Studio software (Version 7.5, Bioinformatics Solutions Inc., Waterloo, Canada). Spectral data were converted to mzXML files using CompassXtract (version 3.1). Peak lists were queried against a UniProtKB/Swiss-Prot (http://www.uniprot.org) *Bos taurus* database using the database search tools PEAKS DE NOVO sequencing, PEAKS BD (database search), PEAKS PTM (post-transitional modifications) and SPIDER (algorithm that matches sequence tags with errors to database sequences for the purpose of protein and peptide identification). For protein identification, the MS and MS/MS error tolerances were set to ±10 ppm and ±0.05 Da, respectively, and one missed tryptic cleavage was allowed. Carbamidomethylation of cysteine was included as a fixed modification, and N-terminal ammonia-loss, deamidation of asparagine and glutamine, phosphorylation of serine and threonine, and oxidation of methionine were set as variable modifications. The false discovery rate was set to 1%.

### Data availability statement

All data generated or analyzed during this study are included in this published article (and its Supplementary Information files).

## Electronic supplementary material

Supplementary Information
