# Supplementary material for: Cattle with a precise, zygote-mediated deletion safely eliminate the major milk allergen beta-lactoglobulin
Source: Sci Rep. 2018 May 16;8:7661. doi: 10.1038/s41598-018-25654-8 (PMC5955954; doi:10.1038/s41598-018-25654-8)
Supplement: Supplementary file 1 — Supplementary Information [file 41598_2018_25654_MOESM1_ESM.pdf]

# Supplementary Information

## Cattle with a precise, zygote-mediated deletion safely eliminate the major milk allergen beta-lactoglobulin

Jingwei Wei, Stefan Wagner, Paul Maclean, Brigid Brophy, Sally Cole, Grant Smolenski, Dan F. Carlson, Scott. C. Fahrenkrug, David N. Wells and Götz Laible

## Supplementary Figures

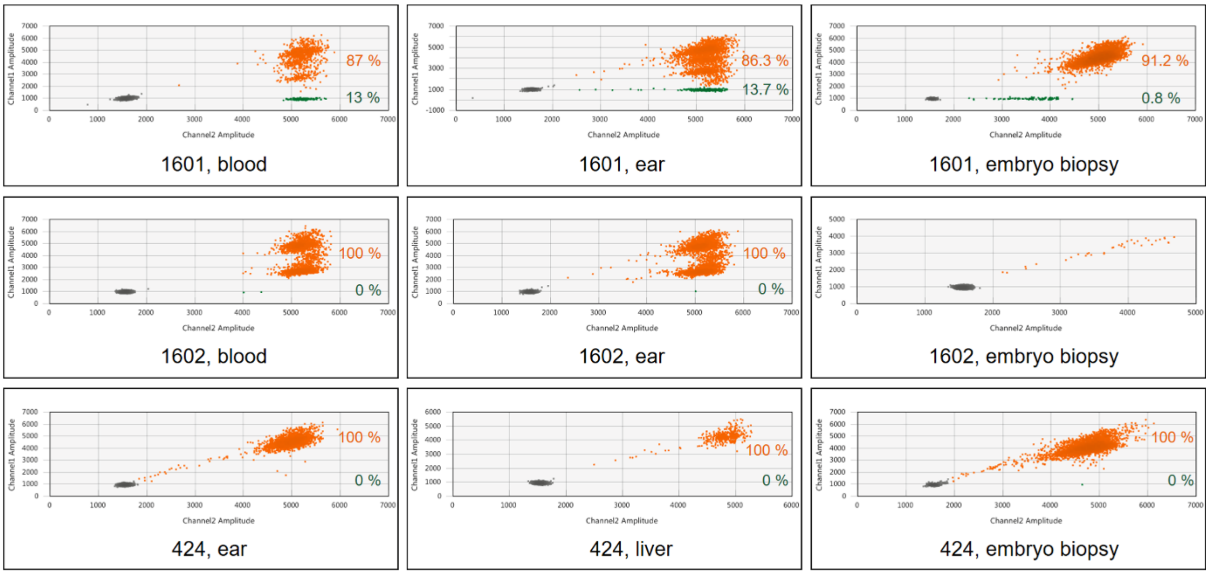


**Supplementary Fig. S1: Assessment of the allelic composition of *LGB* target locus by ddPCR.** Shown are 2D amplification plots with the nine bp deletion HDR probe for the indicated 1601, 1602 and 424 samples. Orange dots represent HDR-positive droplets, green droplets represent wild type and non-HDR indel alleles and black dots represent droplets with no amplification. Contributions of the nine bp deletion and wild type/non-HDR indel alleles are given for each plot.

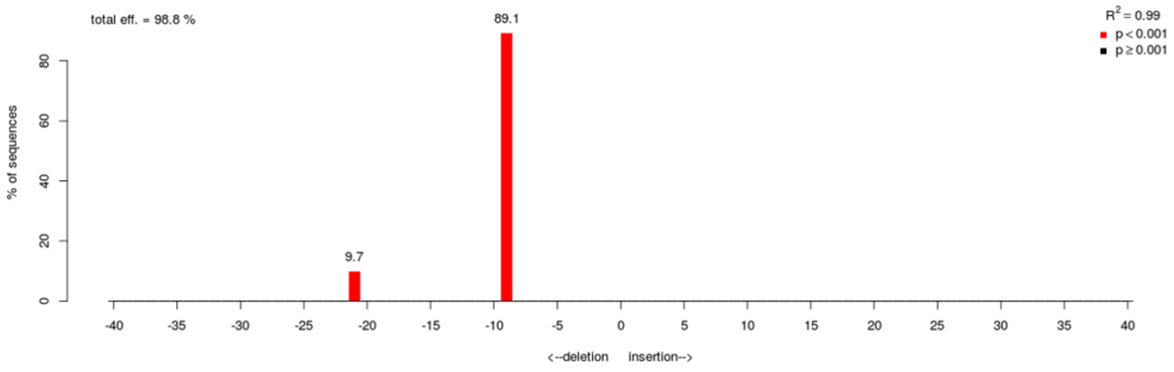


**Supplementary Fig. S2: TIDE analysis.**

Shown are the two allelic variants and their relative quantities predicted by TIDE analysis to be present in the amplified LGB fragment from 1601 genomic DNA isolated from a blood sample.

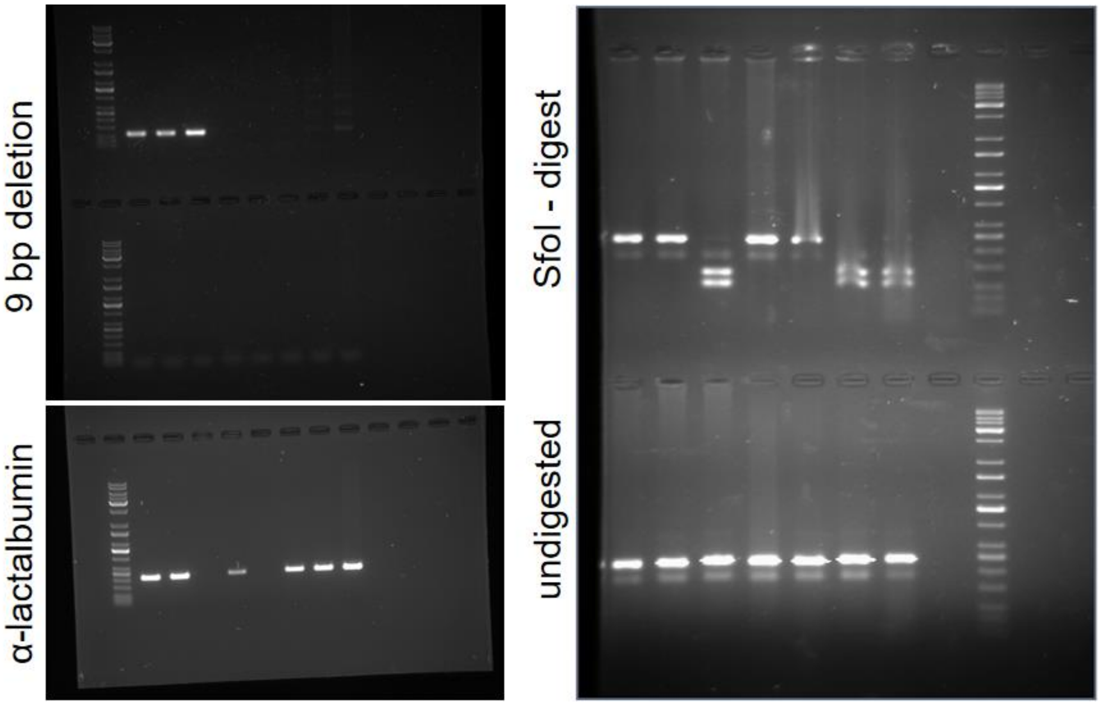


**Supplementary Fig. S3: Full size gel photos used for the cropped images shown in Fig. 3.**

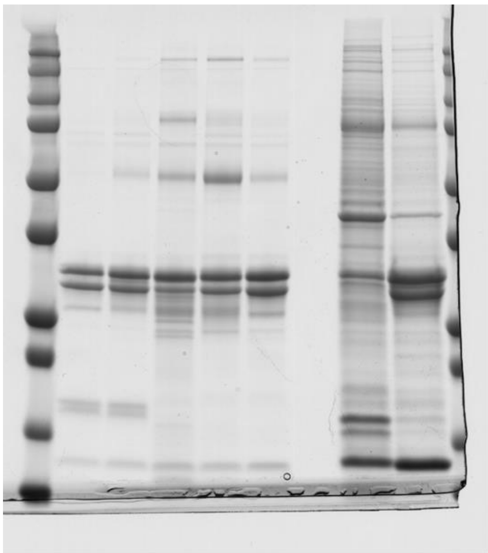

Coomassie

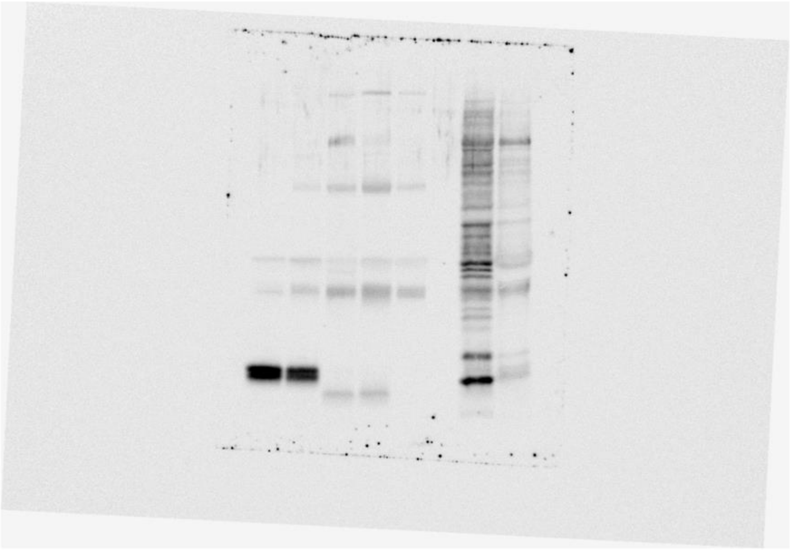

western

**Supplementary Fig. S4: Full size protein gel nd western membrane photos used for the cropped images shown in Fig. 5.**

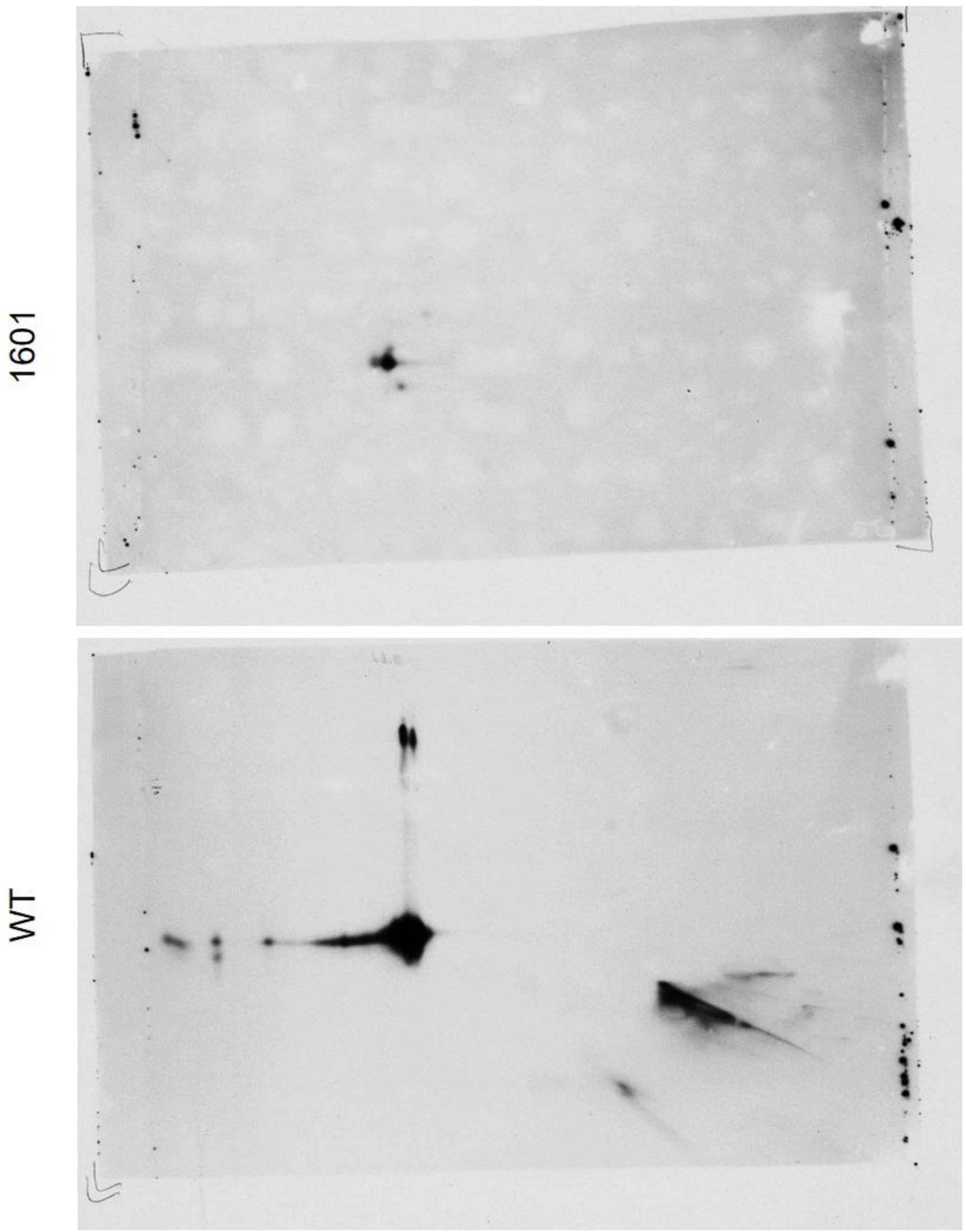


**Supplementary Fig. S5: Full size western membrane photos used for the cropped images shown in Fig. 6A.**

**Supplementary Tables**

**Table S1: Summary of *in vitro* embryo development of IVF zygotes co-injected with TALENs and ODN HDR template.**

| IVF Sire | Injection sessions | TALEN plasmids | ssODN | Zygotes injected | Total embryo development | Development to grade1/2 | nPCR* +ve | nPCR* +ve & TaqMan +ve |
|---|---|---|---|---|---|---|---|---|
| Deacon | 20 | btBLG 1.2 | 986 | 1219 | 452/1219 (37 %) | 193/1219 (16 %) | 64/193 (33 %) | 43/193 (22 %) |
| Reeds Chee | 4 | btBLG 1.2 | 986 | 226 | 83/226 (37 %) | 28/226 (12 %) | 5/28 (18 %) | 1/28 (4 %) |
| Sirocco | 1 | btBLG 1.2 | 986 | 66 | 23/66 (35 %) | 13/66 (20 %) | 6/13 (46 %) | 6/13 (46 %) |
| Total | 25 | | | 1511 | 558/1511 (37 %) | 234/1511 (15 %) | 75/234 (32 %) | 50/234 (21 %) |

* nPCR: nested PCR

**Supplementary Table S2: Quantification of allelic variants present in calves and embryos by deep sequencing.**

| Sample | | Maximum no. of reads per replicate | Wild type allele | 9 bp deletion | 21 bp deletion | 24 bp deletion | 66 bp deletion |
|---|---|---|---|---|---|---|---|
| **Calves** | 1601, blood | 85,470 | 1% | 85% | 13% | 0% | 0% |
| | 1601, ear | 83,754 | 2% | 81% | 17% | 0% | 0% |
| | 1602, blood | 46,928 | 2% | 98% | 0% | 0% | 0% |
| | 424, ear | 66,777 | 1% | 99% | 0% | 0% | 0% |
| | 424, muscle | 70,751 | 1% | 99% | 0% | 0% | 0% |
| | 424, kidney | 56,113 | 1% | 99% | 0% | 0% | 0% |
| | 424, lung | 45,747 | 2% | 98% | 0% | 0% | 0% |
| | 424 , testis | 49,052 | 2% | 98% | 0% | 0% | 0% |
| | 424, liver | 84,551 | 3% | 97% | 0% | 0% | 0% |
| **Embryos** | TS26 (1601) | 80,196 | 8% | 91% | 0% | 0% | 0% |
| | TS62 (1602) | 82,067 | 1% | 99% | 0% | 0% | 0% |
| | TS29 (424) | 65,709 | 56% | 8% | 0% | 32% | 4% |
| | WT1 | 54,025 | 99% | 1% | 0% | 0% | 0% |
| | WT2 | 36,568 | 100% | 0% | 0% | 0% | 0% |
| | WT3 | 75,636 | 100% | 0% | 0% | 0% | 0% |
| | WT7 | 69,058 | 100% | 0% | 0% | 0% | 0% |
| | WT8 | 53,413 | 100% | 0% | 0% | 0% | 0% |

**Supplementary Table S3: Milk composition determined by near infrared analysis.**

| Sample | Cow age | Type of lactation | Time of lactation | Fat* | Protein* | Lactose* | Total Solid* | Solid Non Fat* |
|---|---|---|---|---|---|---|---|---|
| 1601 | 8 months | induced | Day 5 | 3.6 | 5.4 | 3.8 | 14.4 | 10.3 |
| KD | 9.5 months | induced | Day 3 | 4.2 | 5.0 | 4.5 | 15.1 | 10.4 |
| WT | 8 months | induced | Day 3 | 4.7 | 4.2 | 4.8 | 14.6 | 9.7 |
| WTP | > 2 years | natural | Early lactation | 3.7 | 4.2 | 4.4 | 13.0 | 9.4 |

* given as percentage (w/w).

**Supplementary Table S4: Primer sequences**

| Primer | Sequence | Application |
|---|---|---|
| 290 | TCCTCTGCCTCTGCTCCAGT | qPCR, CSN2 locus |
| 291 | CGGACAGGACCGAGTACAGG | qPCR, CSN2 locus |
| 840 | AGGCCTCCTATTGTCCTCGT | Amplification of LGB locus |
| 841 | GCAAAGGACACAGGGAGAAG | Amplification of LGB locus |
| 986 | GCAGCCATGAAGTGCCTCCTGCTTGCCCTGGCCCTCACYTAGGCCCTCATYGTCA CCCAGACCATGAAGGGCCTGGATATCCAGAAGGTTCGAGGGTGSC | Homology repair template |
| 994 | CTGGCCCTCACYTAGGCCCT | Primer specific for 9 bp deletion |
| 1015 | GCAAGACACTAAACGCCTGG | Amplification of LGB locus |
| 1016 | TCGTCGGCAGCGTCAGATGTGTATAAGAGACAGGCTGGCCCTCCTCCTGTATA | Illumina sequencing, LGB locus |
| 1017 | GTCTCGTGGGCTCGGAGATGTGTATAAGAGACAGGCAAGACACTAAACGCCTGG | Illumina sequencing, LGB locus |
| 1048 | CUCACYUAGGCCCUCA | 9 bp deletion TaqMan probe, FAM |
| 1051 | TGCAGCCATGAAGTGCCT | TaqMan PCR primer, LGB |
| 1052 | CACCCTCGAACCTTCTGGATA | TaqMan PCR primer, LGB |
| 1337 | CCTGTATAAGGCCCCGA | ddPCR primer |
| 1338 | CCCTCGAACCTTCTGGATA | ddPCR primer |
| 1339 | CACTTGTGGCGCCCAGGC | Drop-off probe, FAM |

| | | |
|---|---|---|
| 1340 | CTCCACTCCCTGCAGAGCTCAGAA | Reference probe, HEX |
| 1341 | CCTCACTTAGGCCCTCATTGTCACC | HDR probe, FAM |
| 1342 | CCT CAC TTG TGG CGC CCA GGC | Dark probe |
